# Gold nanoparticles assembled with dithiocarbamate-anchored molecular wires

**DOI:** 10.1038/srep15273

**Published:** 2015-10-16

**Authors:** Nini E. A. Reeler, Knud A. Lerstrup, Walter Somerville, Jozsef Speder, Søren V. Petersen, Bo W. Laursen, Matthias Arenz, Xiaohui Qiu, Tom Vosch, Kasper Nørgaard

**Affiliations:** 1 Nano-Science Center & Department of Chemistry, University of Copenhagen, Universitetsparken 5, 2100 Copenhagen, Denmark; 2 Sino-Danish Centre for Education and Research (SDC), Niels Jensens Vej 2, 8000 Aarhus C, Denmark; 3 MacDiarmid Institute for Advanced Materials and Nanotechnology, School of Chemical and Physical Sciences, Victoria University of Wellington, PO Box 600, Wellington 6140, New Zealand; 4 National Center for Nanoscience and Technology, Beijing 100190, P. R. China

## Abstract

A protocol for the bottom-up self-assembly of nanogaps is developed through molecular linking of gold nanoparticles (AuNPs). Two π-conjugated oligo(phenylene ethynylene) molecules (OPE) with dithiocarbamate anchoring groups are used as ligands for the AuNPs. OPE-4S with a dithiocarbamate in each end of the molecule and a reference molecule OPE-2S with only a single dithiocarbamate end group. The linking mechanism of OPE-4S is investigated by using a combination of TEM, UV-Vis absorption and surface enhanced Raman spectroscopy (SERS) as well as studying the effect of varying the OPE-4S to AuNP concentration ratio. UV-Vis absorption confirms the formation of AuNP aggregates by the appearance of an extended plasmon band (EPB) for which the red shift and intensity depend on the OPE-4S:AuNP ratio. SERS confirms the presence of OPE-4S and shows a gradual increase of the signal intensity with increasing OPE-4S:AuNP ratios up to a ratio of about 4000, after which the SERS intensity does not increase significantly. For OPE-2S, no linking is observed below full coverage of the AuNPs indicating that the observed aggregate formation at high OPE-2S:AuNP ratios, above full AuNP coverage, is most likely of a physical nature (van der Waals forces or π-π interactions).

The controlled fabrication of molecular nanogap junctions, where a pair of metallic electrodes is separated by molecular dimensions, is a fundamental challenge in nanoscience, and is currently beyond the capabilities of industrial-scale microfabrication schemes. Such nanogap structures show potential for application in e.g. spectroscopy^1^, plasmonics^2^ and in molecular electronics^3^,^4^. Advanced top-down approaches, such as mechanically controllable break junctions^1^,^5^ and scanning probe methods^6^,^7^ have proven successful in the construction of molecular nanogap junctions^4^,^8^. However, these methods generally suffer from low throughput, lack of scalability and a failure to produce permanent nanogaps. Conversely, molecular-scale nanogaps can potentially be produced in large quantities and high yield from the bottom-up through the use of self-assembly principles^3^,^9^. In particular, the molecular assembly of metallic nanoparticles (NPs) into dimers^10^,^11^ and other structures has been demonstrated as a possible route towards the fabrication of nanogap junctions^3^. Nevertheless, controlled self-assembly of colloidal particles require careful considerations of numerous factors such as solvent effects, pH value and surface properties^12^,^13^,^14^. Small changes to the AuNP surface properties, such as coating with a self-assembled monolayer (SAM) and change of end group functionalities, from e.g. a charged species to a non-polar group, will have dramatic effects on the surface energies and hence the stability of the AuNPs towards aggregation. One particular challenge concerns the bottom-up assembly of molecular nanogap junctions for use in molecular (opto-)electronics. This approach requires that a functional, typically π-conjugated, molecule is inserted into the nanogap, which severely hampers the self-assembly process due to solubility issues^15^,^16^,^17^. In this paper, the effect of changing the ratio of linker to AuNP concentration was investigated. As shown, this ratio can impact the amount and distribution of AuNPs that are linked together. The latter is important for controlling the bottom-up synthesis of linked nanogaps.

Additionally, the method proposed here is a versatile synthetic route for covalent linking of gold nanoparticles (AuNPs) using a π-conjugated oligo(phenylene ethynylene) molecules (OPE). The OPE is terminated in both ends with a dithiocarbamate anchoring group (Fig. 1A)^18^, which has proven to be a good alternative to the commonly used thiol^19^,^20^. The charged nature of the sodium dithiocarbamate group(s) facilitates dissolution of the OPE wires in methanol and therefrom in the aqueous colloid solution, while – at the same time – ensuring a stable covalent binding to the AuNPs. The assembly process is characterized using surface enhanced Raman spectroscopy (SERS) in combination with absorption spectroscopy (UV-Vis), transmission electron microscopy (TEM), and investigated as a function of the ratio between linker molecule and AuNP concentrations.

## Results and Discussions

AuNPs were synthesized in accordance with literature procedures, as described in the materials and methods section. TEM was used to characterize the size and shape of the synthesized AuNPs, revealing a slightly elongated spherical shape as shown in Fig. 2. The dimensions (length) of the major and minor axes of the elongated AuNP spheres were estimated from the TEM micrographs revealing an average value of the major axis of around 73 nm with an aspect ratio of about 1.25. The corresponding histograms of size and aspect ratio distribution are shown in the supporting information (Figure SI1). The average size of the AuNPs was calculated using the method by Haiss *et al.*^21^ to be 71 nm (see Supporting Information). In addition to the size measurements, TEM was applied to study the linking process of AuNPs with OPE-4S and to investigate the presence of nanogaps. Figure 2 shows micrographs of three samples with OPE-4S:AuNP ratios of A: 2000, B: 10000 and C: 100000, respectively. The micrographs clearly reveal the presence of nanogaps between isolated AuNPs, with very uniform gap distances in the 1–1.5 nm range. Additional overview TEM micrographs are shown in Figure SI2. To further study the chemical nature of these nanogaps, we employed a number of spectroscopic techniques, such as UV-Vis absorption and SERS. To prevent effects caused by drying-induced aggregation, these measurements were performed in solution.

UV-Vis absorption is an excellent technique for studying the aggregation of AuNPs in solution, utilizing the position of the AuNP plasmon absorption as an indicator. For molecularly linked AuNPs, the degree of aggregation as well as the distance between the linked AuNPs, may result in the appearance of a new red shifted band. This band in the 700–950 nm region is known as the extended plasmon band (EPB)^22^,^23^,^24^. To estimate the effect of gap distance and chain length on the EPB in linked AuNPs, theoretical calculations where performed for a single AuNP and for AuNP dimers, with gap sizes of 1 and 3 nm (Fig. 3C), as well as for linear AuNP trimers with interparticle distances of 1 and 3 nm (Fig. 3D). For individual AuNPs only the transversal plasmon band (TPB) is observed with a maximum at 535 nm, which is close to the observed experimental value (see Fig. 3A). As the distance between the AuNPs decreases, a red shifted plasmon band appears, exhibiting larger red shifts with smaller gap sizes. For the linear AuNP trimer, the red shifted band red shifts even further compared to the AuNP dimer, as shown in Fig. 3D.

Experimentally, the addition of OPE-4S to a solution of AuNP results in the appearance of an EPB (Fig. 3A,B), where initially only the TPB is observed at 533 nm. The extent of aggregation depends strongly on the ratio between the OPE-4S linker and the AuNPs, with the high ratio (Fig. 3A) producing a more intense EPB compared to the lower ratio situation (Fig. 3B). If two AuNPs are chemically linked by one or more OPE-4S molecules, the gap between them is likely to be equal to or slightly less than the length of the molecule (≈2.5 nm) depending on the orientation of the linking OPE-4S in the gap. Figure 3A shows that the EPB tends to broaden and red shift further over time for a high ratio of 80000, indicating that the AuNPs aggregation increases over time, while for low ratios of 4000 this effect is not observed. The latter suggest that the aggregation process is finished faster at lower ratios compared to higher ratios.

**Figure 1:**
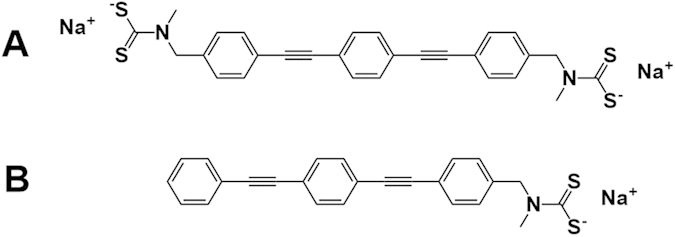
Dithiocarbamate end-capped oligo(phenylene ethynylene) (OPE) compounds used in this study. OPE-4S containing two anchoring groups (**A**), OPE-2S containing one anchoring group (**B**).

To determine how the aggregation mechanism changes with the concentration of OPE-4S, UV-Vis absorption spectra were measured for a large range of OPE-4S:AuNP ratios from below to well above fully covered AuNPs. We estimate the maximal number of OPE-4Ss that can bind to a single AuNP by assuming spherical AuNPs with a diameter of ~71 nm, and a surface area of about 16000 nm^2^. The 71 nm is based on estimating the diameter from the absorption spectrum using the method from Haiss *et al.*^21^ The surface area of a single OPE-2S (containing one dithiocarbamate anchoring group, see Fig. 1B) in densely packed high quality self-assembled monolayers (SAMs) on (flat) Au-surfaces was found by Wei *et al.*^18^ to be 0.42 nm^2^/molecule. This gives an estimated maximum of about 38000 molecules for a full coverage of the AuNPs.

The UV-Vis absorption in Fig. 4A show that from OPE-4S:AuNP ratios of 3000 to 110000, aggregation is present due to the appearance of the EPB. For the low (3000–5500) and intermediate (15000–30000) OPE-4S:AuNP ratios, the intensity of the TPB decreases, while the intensity and red shift of the EPB increases. Even though in the intermediate OPE-4S:AuNP ratio range, the AuNPs starts to be more densely covered, the OPE-4S is still able to aggregate the AuNPs. At higher ratios (55000–110000), a reversal of the trend is observed as can be seen by the lower intensity and reduced red shifted position of the EPB. For intermediate and high OPE-4S:AuNP ratios, a shoulder is observed on the long wavelength side of the TPB, which could be attributed to some of the smaller aggregates as they seem to match with the theoretical calculations of such species (see Fig. 3C,D). The reversal of the trend observed for the high OPE-4S:AuNP ratio range could be due to the fact that the AuNPs are now in the fully covered range, reducing the likelihood of OPE-4S to interdigitate the dense OPE-4S layer on the other AuNP. Additionally, the dithiocarbamate end groups are negatively charged, which will also lead to increased repulsion between two covered AuNPs. However, aggregation still occurs as can be seen in Fig. 4A, although at a reduced speed compared to the low OPE-4S:AuNP ratios (see Fig. 3). Furthermore, the more pronounced shoulder in the TPB band for the highest mixing ratios could indicate the presence of a higher relative number of smaller aggregates.

The UV-Vis absorption results suggest that the OPE-4S:AuNP ratio can be used to influence the nature of formed AuNP aggregates. As a control, we performed similar experiments using the molecule OPE-2S (see Fig. 1B), which contains a single dithiocarbamate linker group on one end of the molecule and an aromatic group on the other end. The results of the UV-Vis absorption measurements using OPE-2S can be seen in Fig. 4B. Only for high OPE-2S:AuNP ratios above 30000 (close to full coverage), an EPB can be observed, indicating some form of aggregation. The intensity and red shift of the EPB keeps increasing with increasing OPE-2S:AuNP ratio from 30000 to 100000. This trend is different from what we observed for OPE-4S, where at high ratios a reversal in intensity and red shift of the EPB band was observed. However, for OPE-2S, no direct molecular linking should be expected as only one anchoring group is present. Therefore, we propose that the aromatic end-groups, at high coverage, will interact by either van der Waals^25^ or π-π interactions^26^, leading to aggregation of the covered AuNPs. This linking mechanism is different than the molecular linking proposed for the OPE-4S.

While UV-Vis absorption measurements can provide information on AuNP interactions and aggregation, surface enhanced Raman spectroscopy (SERS) can give a direct chemical signature from the molecular linker, and reveal if it is absorbed onto the AuNP surface and potentially present in the nanogap between the particles (SERS “hot spot”)^27^,^28^.

All SERS measurements were performed in solution, providing a proper read-out of the actual AuNP aggregates, while preventing issues related to drying-induced aggregation. SERS experiments were performed using 633 nm or 785 nm laser light. Figure 3A shows the relation between the plasmon bands and the laser wavelength. Both lasers can probe the EPB and the 633 nm laser can also probe the TPB. Since the signal from molecules inside a nanogap is expected to be substantially larger (due to the SERS enhancement in the hot spots)^27^,^29^, we assume that most of the Raman signal observed from either experiment arises from the OPE-4Ss inside the nanogap between the AuNPs (see Figure SI5 in the Supporting Information).

Figure 5A shows the bulk Raman spectra of OPE-4S and citrate, as well as a SERS spectrum of OPE-4S linked AuNPs. The bulk Raman spectrum of OPE-4S displays three main peaks. The peaks at 1133 cm^−1^ (peak 1) and 1601 cm^−1^ (peak 2) arise from the benzene groups^30^, while the peak at 2211 cm^−1^ (peak 3) is associated with the acetylene groups^31^. These three major peaks are also observed in the SERS spectrum for the linked AuNPs, although the relative intensities of the peaks are different in the SERS spectrum compared to the bulk OPE-4S sample. The broad peak from 3200–3600 cm^−1^ in the SERS spectrum is from water in the solution.

The SERS enhancement factor was determined by measuring the Raman signal from a solution of 0.36 mM OPE-4S in MeOH and the SERS signal from a solution containing 3.6 μM OPE-4S and 30 pM AuNPs as seen in Fig. 5B. The two spectra were measured under exactly the same conditions with an exposure time of 60 seconds using a 785 nm laser. The analytical enhancement factor (AEF), as described by Le Ru *et al.*^32^ is given in equation 1 and represents the increase of the OPE-4S signal in the SERS measurements versus the normal Raman condition.
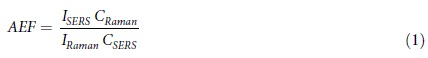
In equation 1, *C*_*SERS*_ and *C*_*Raman*_ are the concentrations of the OPE-4S in the solutions with and without AuNPs, respectively. *I*_*SERS*_ and *I*_*Raman*_ are the intensity of the measured Raman spectrum with and without AuNPs, respectively.

The intensities were determined by fitting a Lorentz function to the specific peaks in Fig. 5B. An AEF value of 5600 was found for this particular OPE-4S:AuNP ratio of 120000 for peak 2 and an AEF value of 2400 for peak 3. Due to the overlap with a methanol peak it was not possible to accurately determine the AEF value for peak 1. The AEF enhancement values might seem small compared to the usual reported enhancement values of ~10^11^ or more for molecules in hot spots^27^. However, the AEF value reflects the average enhancement of all the molecules in the solution where most of them are presumably not present inside the hot spots^32^. The high OPE-4S:AuNP ratio therefore decreases the AEF value. Figure SI8 in the Supporting Information shows that lower OPE-4S:AuNP ratios result in much higher AEF values.

To check if the SERS signal of the linked OPE-4S:AuNP solution is linear with concentration, a dilution series was produced from a solution with an OPE-4S:AuNP ratio of 4000 (the ratio stays constant in the dilution). Figure 6A shows the SERS intensity of the three major peaks in the SERS spectrum as a function of the AuNP concentration, fitted with a linear function.

Next, the effect on the SERS signal of varying the OPE-4S:AuNP ratio was investigated. SERS measurements were performed within a timeframe of 10 to 40 minutes after preparation in order to limit the potential influence of precipitation at large OPE-4S:AuNP ratios. The relatively large error bar, representing the standard deviation of all the measurements, originates from the fact that only a small volume is probed with our diffraction limited confocal microscope. Figure SI10 shows the fluctuations from frame to frame representing the stochastic fluctuations of SERS active AuNPs/aggregates diffusion through the probing volume. In Fig. 6B, the SERS intensities of the three major peaks are depicted for OPE-4S:AuNP ratios ranging from 0–8000. We observed a steady increase in SERS intensity up to a ratio about 4000. A similar trend was also found when using the 785 nm laser setup (see Figure SI8 and Figure SI9A). At ratios above 4000, the SERS signal levels off as shown in Fig. 6B. Even at much higher OPE-4S:AuNP ratios than 8000 (see Fig. 6C and Figure SI8 and Figure SI9) and up to about 180000, the SERS signal does not increase further as it did in the regime from 0–4000. The exact reason why the SERS signal levels off at OPE-4S:AuNP ratios above 4000 is not fully understood, but one could speculate that the number of active SERS hot spots does not increase much above a mixing ratio of 4000. Although the UV-Vis absorption data shows that the aggregation increases up to a ratio of 30000, this could mean that the number of AuNP per aggregate increases, but not necessarily the number of AuNP hotspots probed using polarized laser light.

Additionally for OPE-2S, the SERS signal was measured as a function of the OPE-2S:AuNP ratio and the results are represented in Fig. 6D. In contrast to OPE-4S, no significant SERS signal was observed in the low ratio regime (up to a ratio of 50000). Only at the highest ratio (100000) a noticeable SERS signal was observed (see Figure SI12 for an example of SERS spectra). The SERS data is in line with the UV-Vis absorption data, where aggregation was observed only at high ratios (100000). Intriguingly, at a ratio of 50000, the UV-Vis absorption spectrum shows aggregation, but no significant SERS signal was observed. It is unclear why no significant SERS signal was observed for the ratio of 50000. A possible explanation could be that the gap size between the AuNPs is larger in these aggregates and therefore a larger number of AuNPs within one aggregate is required to see a detectable SERS signal^29^.

## Conclusion

An OPE molecule with two dithiocarbamate anchoring groups was used to study the linking of AuNPs suspended in water. Based on TEM, UV-Vis absorption and SERS results, we propose different linking regimes. At low OPE-4S:AuNP ratios the AuNPs are not fully covered and can directly link two AuNPs through the two dithiocarbamate end groups, resulting in the appearance of an EPB. From a ratio of 4000 to full coverage (calculated to occur around 38000), an increase in aggregation through the direct linking of the AuNPs is derived from the UV-Vis absorption spectra through an intensity increase and further red shift of the EPB. For ratios above the full coverage regime, the EPB drops in intensity and the red shift is less. The latter could indicate that at high OPE-4S:AuNP ratio, the linking is more difficult, but since the covering and linking is time dependent, some molecular linking can occur before the AuNPs are fully covered, perhaps limiting the aggregates to smaller numbers (hence the reversal in the EPB trend). The reason for this assumption is that full coverage makes interdigitation much harder and the negatively charged dithiocarbamate end groups will also tend to repel each other. The proposed model, based on the OPE-4S results, was tested by conducting similar UV-Vis absorption experiments for OPE-2S which only has one dithiocarbamate anchoring group, preventing direct molecular linking between two AuNPs. At OPE-2S:AuNP ratios below full coverage no significant EPB signal was observed. At high ratios above full coverage, we propose that aggregation occurs due to the van der Waals or π-π interactions of the outer aromatic units. These differences between the two OPE molecules were also observed in the SERS experiments. The results presented here shed more light on the effect of the OPE:AuNP ratio on AuNP aggregation and hints to the presence of different linking regimes. We showed for OPE-2S that aggregation can still be observed for very high ratios (above full coverage) even though molecular linking is not possible.

## Material and Methods

### AuNPs Synthesis

**Figure 2:**
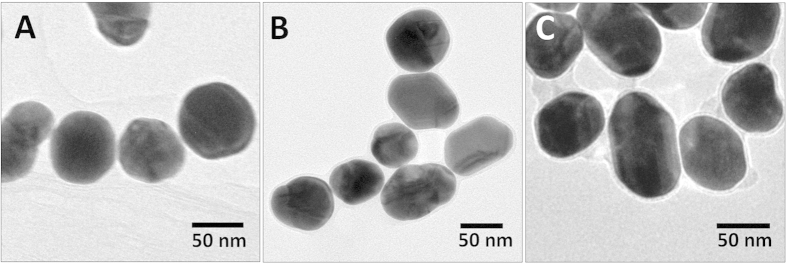
TEM micrographs from dried solutions of AuNPs mixed with OPE-4S in three different ratios 2000 (A), 10000 (B) and 100000 (C).

**Figure 3:**
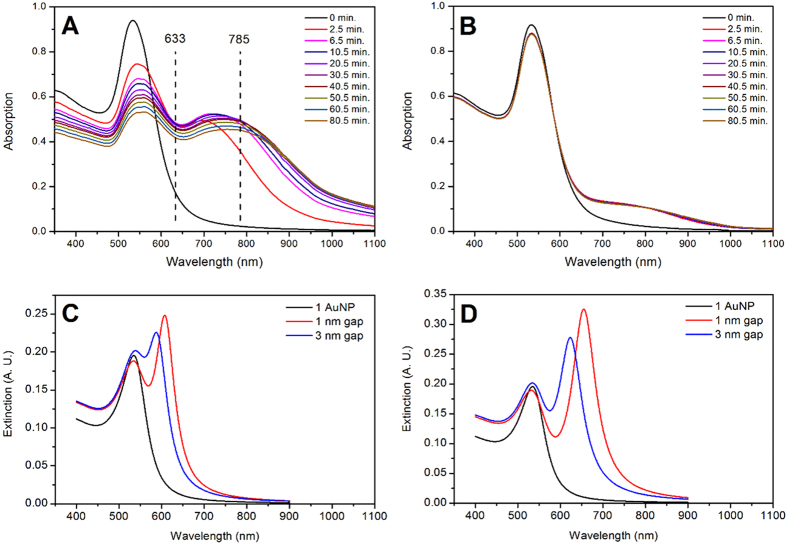
UV-Vis absorption spectra of AuNPs mixed with OPE-4S in ratios of 80000 OPE-4S:AuNP (A) and 4000 OPE-4S:AuNP (B). Simulated UV-Vis absorption spectra of linked 50 nm spherical AuNP dimers (**C**) and linear AuNP trimers (**D**) with different gap sizes. For the AuNP dimer calculations, the red shifted band was found at 609 and 587 nm for the 1 and 3 nm gap, respectively and for the AuNP trimer at 655 and 623 nm.

**Figure 4:**
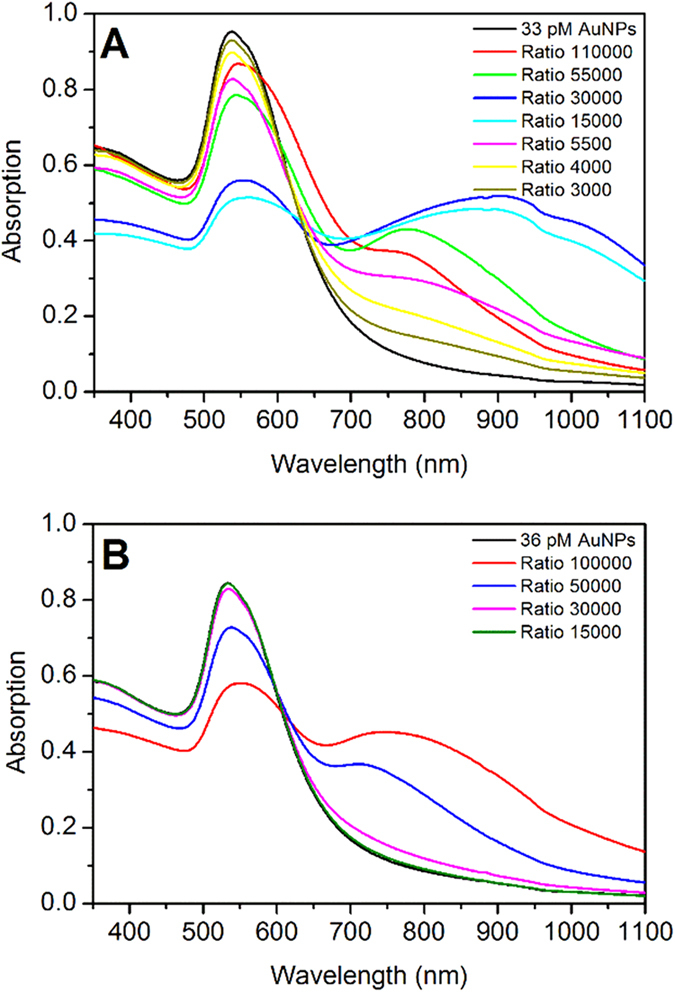
Absorption spectra of different ratios of OPE-4S:AuNP with 33 pM AuNP (A) and of different ratios of OPE-2S:AuNP (only one dithiocarbamate end group) with 36 pM AuNPs (B). Each spectrum is measured 10 min. after the addition of the OPE-4S.

The AuNPs were synthesized by the method of Frens^33^. In short a solution of 50 mL 0.25 mM HAuCl_4_·3H_2_O in ultra pure water (milliQ) is heated to the boiling point followed by the addition of 0.30 ml 34 mM Na_3_-Citrate (HOC(COONa)(CH_2_COONa)_2_·2H_2_O) in milliQ. After about 2 min the solution turned dark blue/purple and then after an additional 5 min red/violet^34^. The heat was turned off, but the round bottomed flask was kept in the hot oil bath or heating plate until room temperature was reached. After this, the solution was stored in the fridge at 4–5 °C. The AuNPs can be stored for months at ~4 °C^35^, though AuNPs were not used if they were much older than a month. The specific volume of citrate-solution should give a AuNP size of about 71.5 nm. Practically, we achieved varying results of the average AuNP size from batch to batch in the range of 50–75 nm in diameter with a concentration of 0.0180 to 0.0451 nM respectively. The AuNP diameter and concentration of the AuNPs were both estimated from the UV-Vis absorption spectra^21^. The formulas used can be found in the Supporting Information. Furthermore, the AuNPs were not perfectly spherical as seen in Fig. 2, but were slightly elongated with an aspect ratio of about 1.25.

The AuNPs were linked by an oligo(phenylene ethynylene) (OPE-4S) with a dithiocarbamate in each end of the molecule as seen in Fig. 1. The first challenge was to actually link the OPE-4S as this organic molecule is only solvable in organic solvents and not in water. However, OPE-4S is solvable in ≥99% HPLC-pure methanol (MeOH) in small amounts, which is mixable with water. 1 mg of the OPE-4S was dissolved in 5 ml of MeOH. When adding a small amount of the OPE-4S in MeOH to the citrate stabilized AuNPs in water, the OPE-4S linked to the AuNPs by exchanging the citrate on the surface instead of immediately falling out of the solution. A series of different concentrations of OPE-4S dissolved in MeOH were produced. 0.010 ml of the specific OPE-4S solution was added to 0.990 ml of the citrate stabilized AuNPs in water and shaken. The same procedure was followed for OPE-2S, though due to solubility difficulties the OPE-2S was dissolved in EtOH instead of MeOH.

### Ultraviolet-visible Absorption Spectroscopy (UV-Vis)

UV-Vis absorption spectra were recorded on a Perkin-Elmer, Lambda 2 spectrophotometer in single beam mode, where data were collected and corrected for background using home-written software, and a Perkin-Elmer, Lambda 950 UV/Vis/NIR spectrophotometer.

### Raman and Surface Enhanced Raman Spectroscopy (SERS)

**Figure 5:**
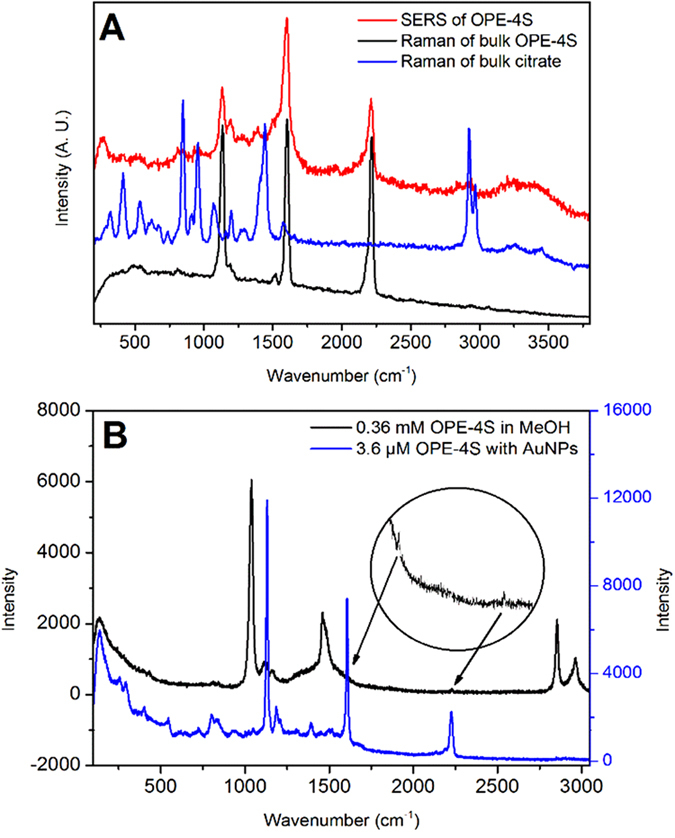
SERS spectrum of AuNPs in a 4000 OPE-4S:AuNP ratio solution (Red) and Raman spectra of bulk OPE-4S (Black) and bulk citrate (Blue), all measured with a 633 nm laser (A). SERS enhancement using a 785 nm laser and a ratio of 120000 OPE-4S:AuNP for the SERS spectrum (**B**).

**Figure 6:**
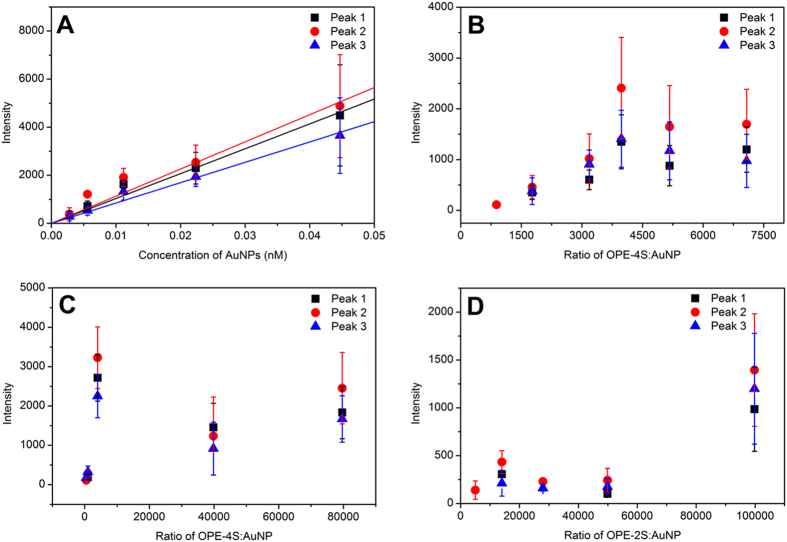
The intensities of the three main peaks in the SERS spectra of OPE-4S as a function of AuNP concentration for a OPE-4S:AuNP ratio of 4000 (A) and the intensities of the same three peaks in the SERS spectra of OPE-4S in samples containing different ratios of OPE-4S:AuNP with AuNP concentrations of 25 pM (B) and 45 pM (C) respectively. The intensities of the three main peaks in the SERS spectra of OPE-2S in samples containing different ratios of OPE-2S:AuNP with 36 pM AuNPs (**D**). A 633 nm laser was used for all the experiments. Each point is the average of five measurements, each measurement being the sum of 100 spectra of 1 second integration time.

For the 633 nm experiments, SERS was performed by pouring the 1 ml sample into a cleaned box with a cover slip in the bottom (Figs 5A and 6B,D). In Fig. 6A,C a big drop was placed directly on a clean cover slip. The Raman measurements for the bulk OPE-4S were performed by placing some powder on a clean cover slip. The box or the cover slip were placed on an Olympus IX71 microscope aligned with a 632.8 nm HeNe CW laser (Thorlabs HRR170-1). A narrow bandpass filter centered at 633 nm (Semrock LL01-633-25) was used to spectrally clean the laser source. In the microscope the laser light was reflected on a dichroic mirror (Semrock LP02-633RU-25) towards a 100 × 1.3 NA immersion oil objective (Olympus UplanFL N) that focused the laser on the sample and collected the Raman signal. A 633 nm longpass filter (Semrock LP02-633RU-25) was used to block the 632.8 nm laser light in the detection path. The Raman spectrum was recorded by using a PI Acton SpectraPro SP-2356 polychromator (300 g mm^−1^ blazed at 500 nm) and a PI Acton SPEC-10:100B/LN_eXcelon Spectroscopy System with a back-illuminated CCD chip (1340 × 100 pixels). The power of the laser focused at the sample was 750 μW (271 kW/cm^2^). X-axis calibration of the spectra was performed after the measurements using a toluene Raman spectrum and a Neon pencil calibration lamp (ORIEL instruments, 6032 neon lamp). 100 spectra were recorded, each 1 second integration time and were summed into one spectrum for analysis.

Raman and SERS measurements were also performed on a commercial dispersive Renishaw inVia plus Raman microscope with a 785 nm excitation source and a 1200 g mm^−1^ grating. The camera is a NIR enhanced CCD camera part no. A-9803-0413. A 50x objective lens was used, which provided a focus point with a diameter of about 2 μm. The spectroscopic resolution is about 1 cm^−1^ and maximum excitation power was used for all the measurements.

100 spectra were recorded, each spectrum in three steps for which each step had an integration time of 1 second. The 100 spectra were summed into one spectrum for analysis. Some of the Raman measurements with the 785 nm laser were performed on a Senterra Raman optical microscope from Bruker. Here an excitation source of 785 nm with a power of 100 mW was used and a 400 g mm^−1^ grating, which gave a resolution of about 9–18 cm^−1^. The camera is a TE-cooled CCD camera. A focus point of about 1 μm was provided by a 50x objective lens. 100 spectra were recorded, each 1 second integration time and were summed into one spectrum for analysis.

### Transmission Electron Microscopy (TEM)

The samples were placed on 300 mesh, thin carbon film supported Cu TEM-grids and characterized by TEM using a Tecnai T20 G2 (Philips FEI, Oregon, USA) equipped with a thermionic electron gun operated at 200 keV. The micrographs were acquired using a Gatan2K UltraScan 1000 CCD camera.

### Theoretical Modeling

The calculations were performed by using the Multiple Sphere T-Matrix (MSTM, v3.0) codes of Mackowski^36^, available for download on the internet^37^, in the case of linear chains of spheres (two or three spheres). The dielectric function of gold was modeled using the method of Etchegoin *et al.*^38^, using a Drude model with interband transitions. Calculations of the extinction efficiency (normalized to the cross-section area of one of the spheres) of gold spheres in water (n = 1.33) averaged over all incident orientations were performed. The simulation code ensures that the results have converged to a specified relative precision (10^−6^) with respect to the order of terms in the series expansions used in the method.

### Supporting Information

Histograms of the size variation of the synthesized AuNPs imaged by TEM, TEM images of three different OPE-4S:AuNP ratios, time evolution of the UV-Vis absorption of AuNPs linked by two different OPE-4S:AuNP ratios, size and concentration calculations of the AuNPs from the UV-Vis absorption spectra, Raman/SERS of mono dispersed citrate stabilized AuNPs, comparison of SERS spectra using 633 and 785 nm laser on similar OPE-4S:AuNP ratios, information on the SERS data analysis using a home written Matlab 2013b program, additional SERS results for the dilution series, additional SERS results for different OPE-4S:AuNP ratios using the 785 nm laser, examples of some of the individual SERS-spectra using the 633 nm laser.

## Additional Information

**How to cite this article**: Reeler, N. E. A. *et al.* Gold nanoparticles assembled with dithiocarbamate-anchored molecular wires. *Sci. Rep.* **5**, 15273; doi: 10.1038/srep15273 (2015).

## Supplementary Material

Supplementary Information
