# Supplementary material for: Gold nanoparticles assembled with dithiocarbamate-anchored molecular wires
Source: Sci Rep. 2015 Oct 16;5:15273. doi: 10.1038/srep15273 (PMC4607996; doi:10.1038/srep15273)
Supplement: Supplementary Information [file srep15273-s1.doc]

**Supporting Information:**

Gold nanoparticles assembled with dithiocarbamate-anchored molecular wires

Nini E. A. Reeler^a,b^, Knud A. Lerstrup^a^, Walter Somerville^c^, Jozsef Speder^a^, Søren V. Petersen^a^, Bo W. Laursen^a^, Matthias Arenz^a^, Xiaohui Qiu^d^, Tom Vosch^a^ and Kasper Nørgaard^a^*

^a^Nano-Science Center & Department of Chemistry, University of Copenhagen, Universitetsparken 5, 2100 Copenhagen, Denmark.

^b^Sino-Danish Centre for Education and Research (SDC), Niels Jensens Vej 2, 8000 Aarhus C, Denmark.

^c^MacDiarmid Institute for Advanced Materials and Nanotechnology, School of Chemical and Physical Sciences, Victoria University of Wellington, PO Box 600, Wellington 6140, New Zealand

^d^National Center for Nanoscience and Technology, Beijing 100190, P. R. China.

**Histograms of the size variation of the synthesized AuNPs imaged by TEM:**


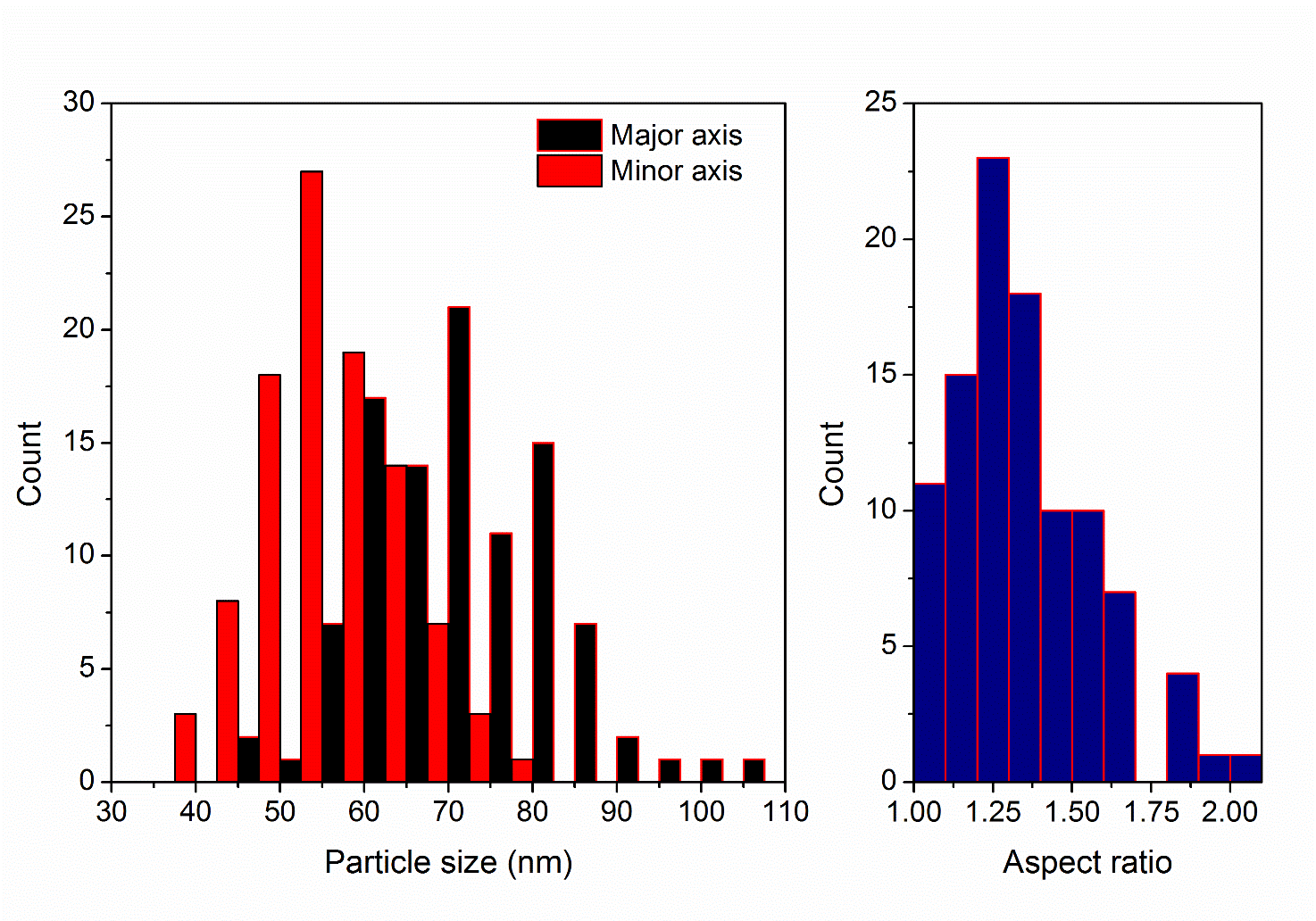


**Figure SI1.** Histogram of the major and minor axis and a histogram of the aspect ratio measured from the TEM pictures of the synthesized AuNPs. In total 100 AuNPs were analysed.

**TEM images of three different OPE-4S:AuNP ratios:**

**
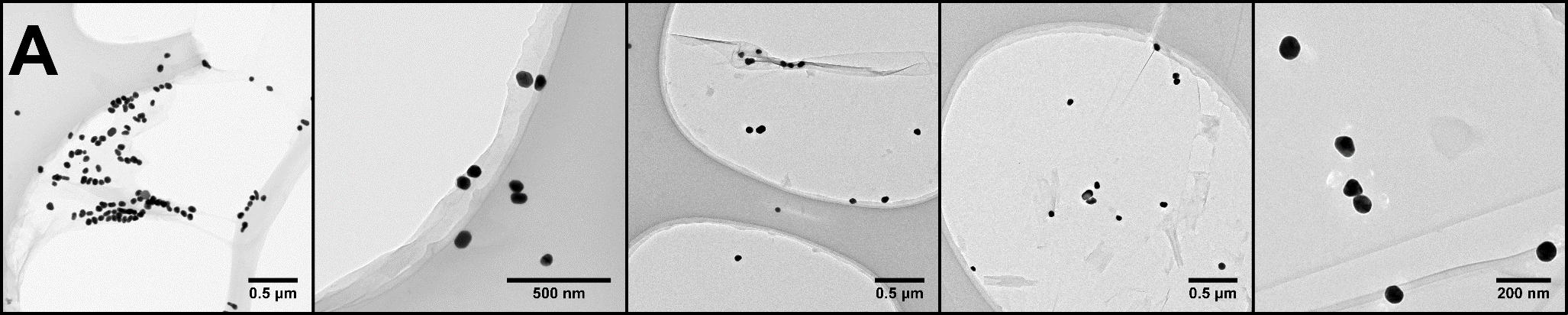
**

**
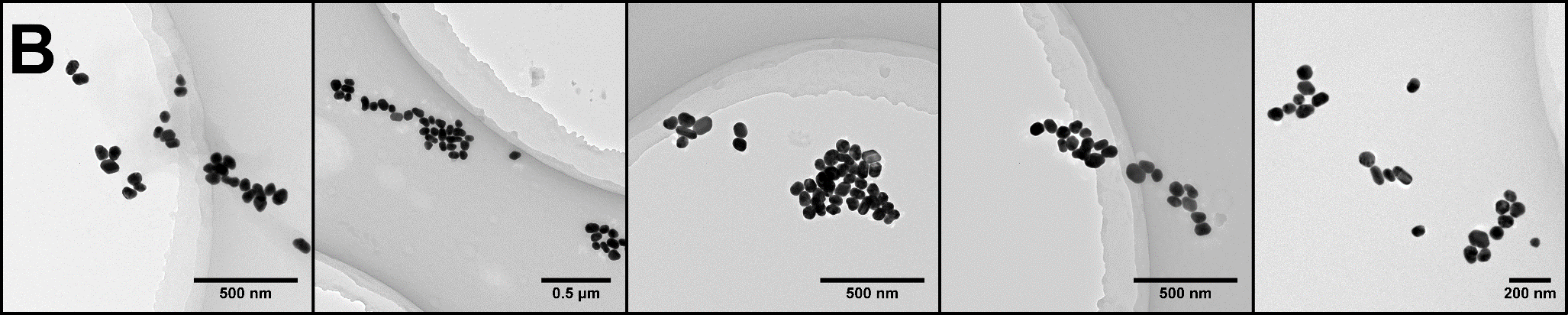
**

**
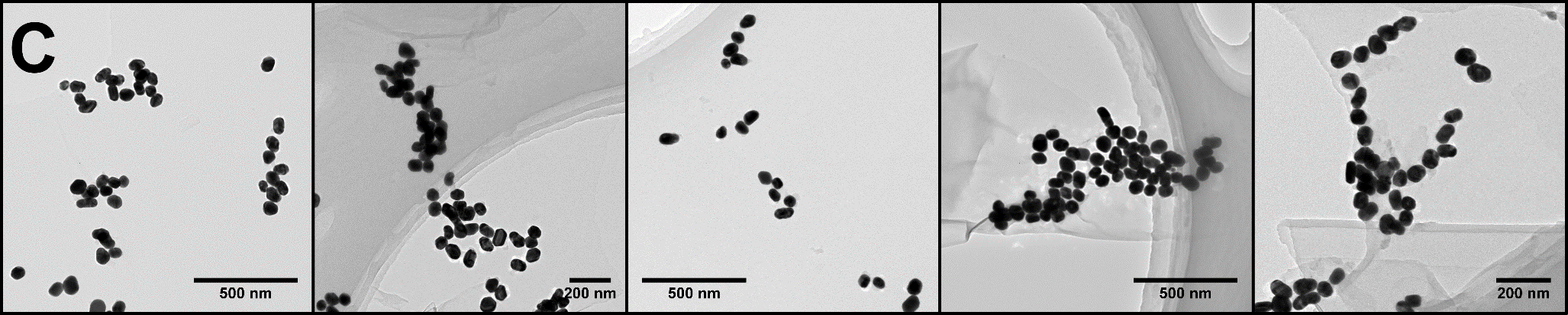
**

**Figure SI2.** TEM images from three different OPE-4S:AuNP ratios A) has a ratio of 2000, B) 10000 and C) 100000.

**Time evolution of the UV-Vis absorption of AuNPs linked by two different OPE-4S:AuNP ratios:**


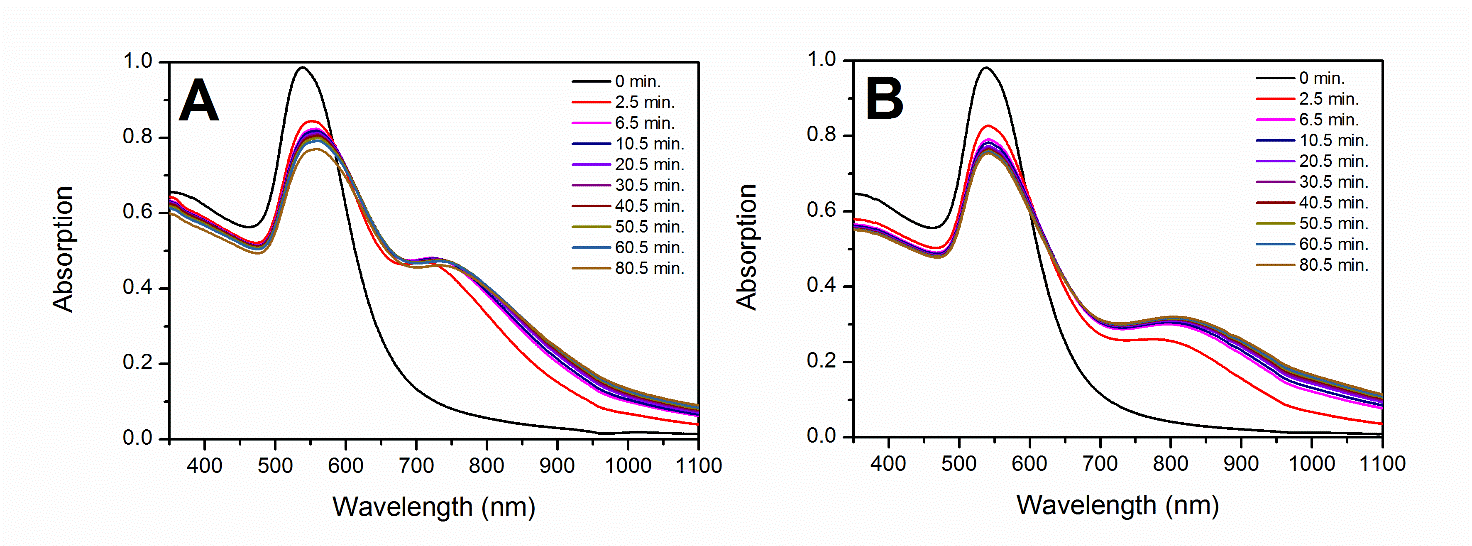


**Figure SI3.** Time evolution of the absorption spectra of OPE-4S linked AuNPs (25 pM ~66 nm diameter) with OPE-4S:AuNP ratios of 150000 (A) and 7000 (B).

**Size and concentration calculations of the AuNPs from the UV-Vis absorption spectrum:**

The size and concentration of the AuNPs were determined using the method by Haiss et al.. The AuNP diameters were calculated using the following equation:


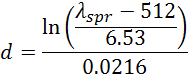


The concentration was then found the equation:


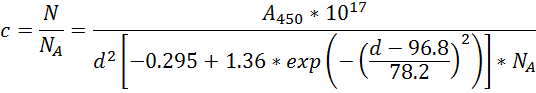


**Raman/SERS of mono dispersed citrate stabilized AuNPs:**


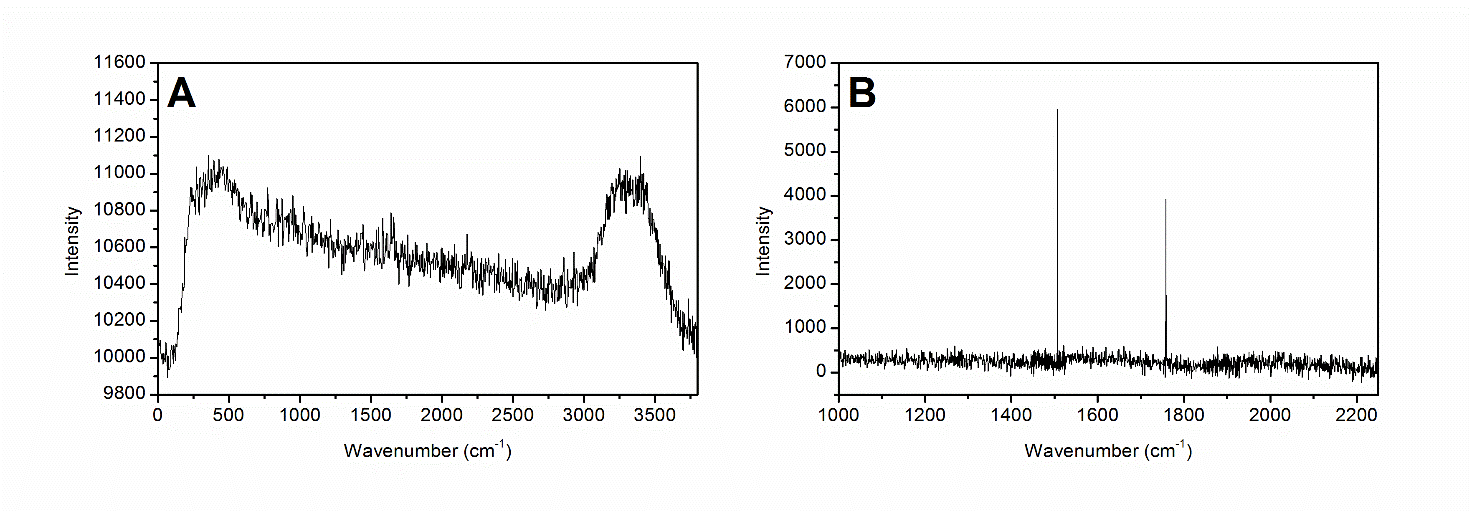


**Figure SI4.** A Raman/SERS measurement of mono disperse citrate stabilized AuNPs measured under the same conditions as the other spectra in the paper using the 633 nm laser (A) and the 785 nm laser (B).

Almost no SERS signal is observed from the citrate on the surface of the mono dispersed AuNPs only a broad Raman peak from 3200-3500 cm^-1^ from water is present for the 633 nm laser (Figure SI4A). For the measurement using the 785 nm laser in Figure SI4B no significant signal was detected. The two narrow spikes around 1500 and 1750 cm^-1^ were due to cosmic rays.

**Comparison of SERS spectra using 633 and 785 nm laser on OPE-4S:AuNP ratios of 3000 and 2000, respectively:**


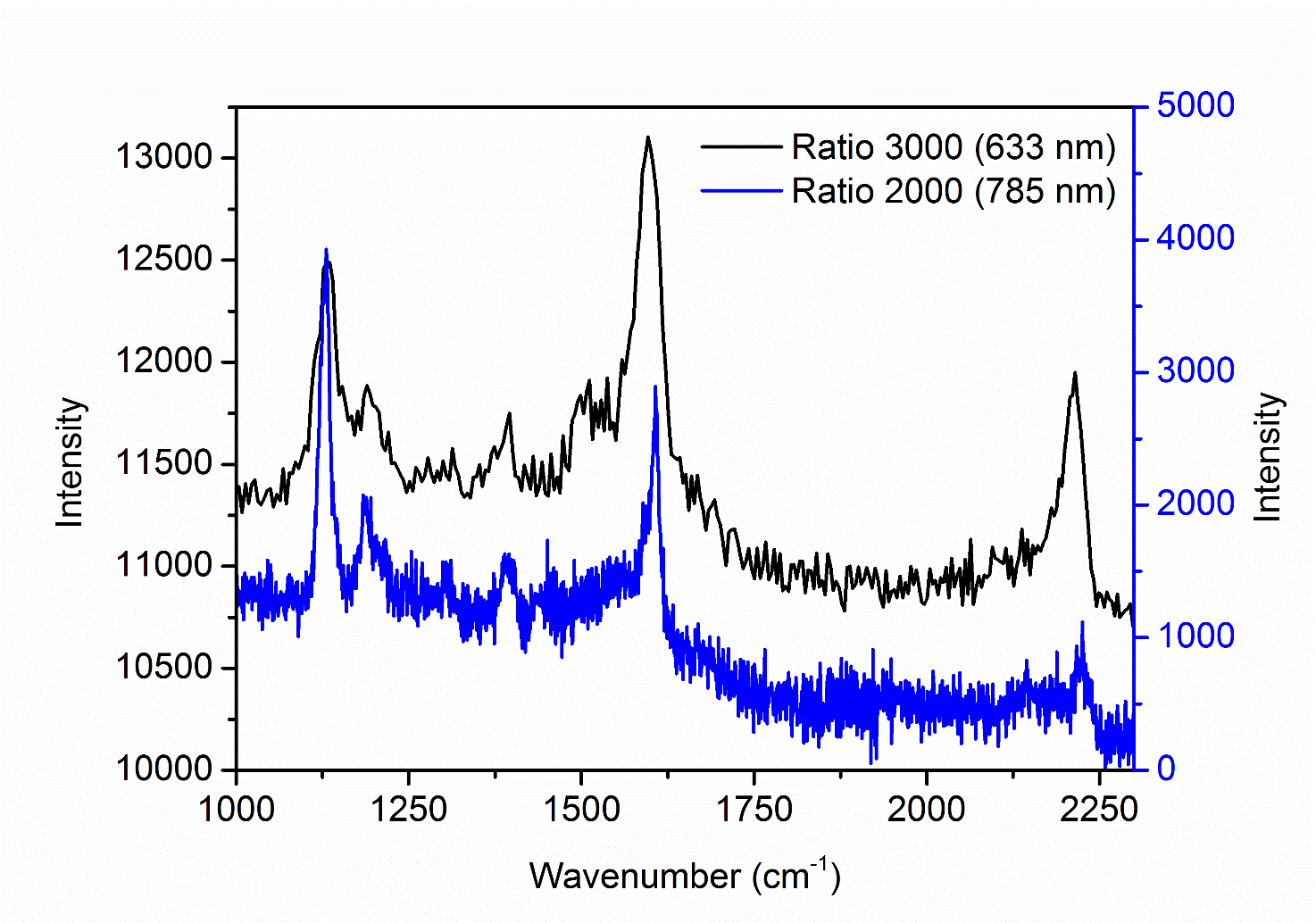


**Figure SI5.** Comparison of SERS-spectra using the two different laser sources.

**SERS data analysis using a home written Matlab 2013b program:**

The SERS spectra, using the 785 nm laser, were measured in three steps with each step having an exposure time of 1 second. This was repeated 100 times. The data collected was analyzed by adding the 100 measured spectra into one spectrum, followed by a background subtraction. For the 633 nm laser no background subtraction was performed. The height was determined by fitting of a Lorentzian function to the three individual peaks; from 1100 to 1160 cm^-1^ for peak 1, from 1570 to 1640 cm^-1^ for peak 2 and from 2190 to 2250 cm^-1^ for peak 3. A similar procedure was followed for the data obtained with the 633 nm laser. However, the individual spectra were measured in one step with an exposure time of 1 second, which was repeated 100 times. After summation of the 100 spectra no background subtraction was performed. The Lorentzian was fitted from 950 to 1300 cm^-1^ for peak 1, from 1450 to 1700 cm^-1^ for peak 2 and from 2100 to 2300 cm^-1^ for peak 3.


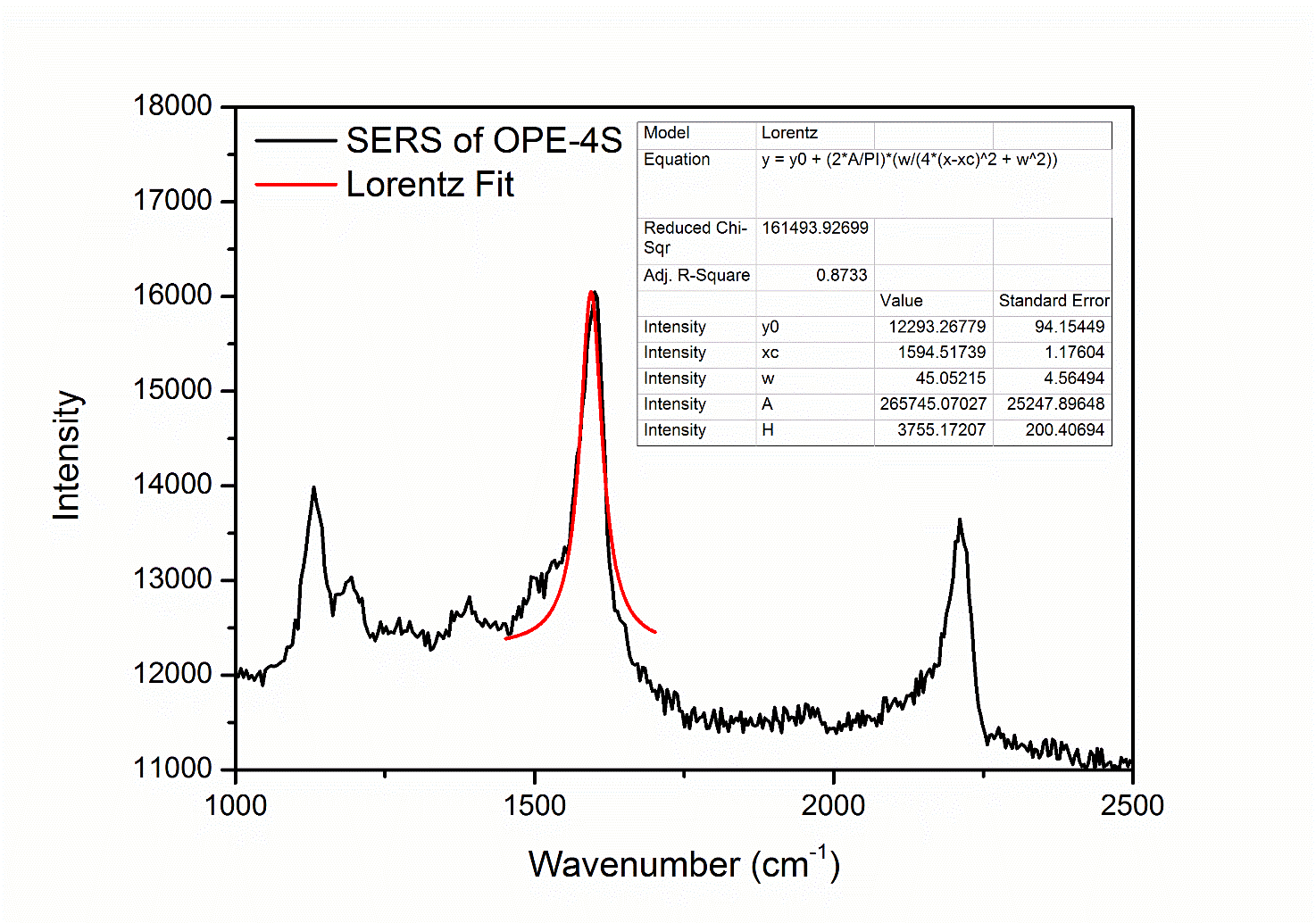


**Figure SI6**. Lorentzian fit to peak 2 in the SERS spectrum of the OPE-4S using the 633 nm laser.

**SERS results for the dilution series:**

The SERS spectra using the 785 nm laser were measured in three steps with each step having an exposure time of 1 second. This was repeated 100 times. The data collected was analyzed by adding the 100 measured spectra into one spectrum. The final spectrum was analyzed as stated above by fitting of a Lorentzian function. From this fit the height of the peak was determined. The same procedure was followed using the 633 nm laser, though five times 100 spectra were collected instead of just three times. Since each spectrum using the 633 nm laser was collected in one step of 1 second more spectra could be collected within the timeframe of 10 to 40 minutes after the addition of the linking OPE-4S.


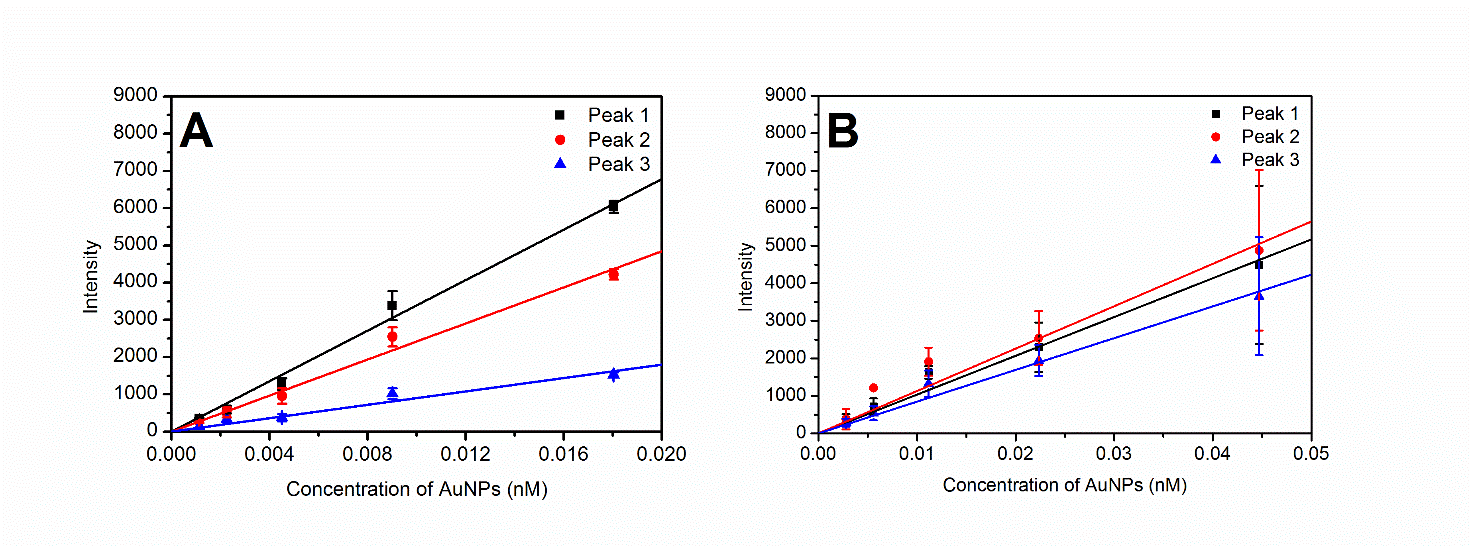


**Figure SI7.** Peak intensities of the three main peaks in the SERS spectra of OPE-4S in a sample of 18 pM AuNPs with the ratio 10000 OPE-4S:AuNP (A) and a sample of 45 pM AuNPs with a ratio of 4000 OPE-4S:AuNP (B) and four dilutions in steps of a 50 % factor for both. The excitation source used for A was 785 nm and for B 633 nm.

In Figure SI6A the fit for Peak 1 has a R^2^ = 0.995, for Peak 2 R^2^ = 0.992 and for Peak 3 R^2^ = 0.977 using the 785 nm laser. In Figure SI6B the fit for Peak 1 has a R^2^ = 0.989, for Peak 2 R^2^ = 0.982 and for Peak 3 R^2^ = 0.989 using the 633 nm laser. In both cases the observed intensity seems to linearly increase with the AuNP concentration. However, the reproducibility for each point using the 633 nm laser is not as accurate as for the 785 nm laser. This is likely due to a smaller focal point as the 100x objective was used for the 633 nm laser instead of the 50x objective used for the other measurements. This was also seen during the measurements where each of the 100 spectra for each point showed a signal almost every time for the 785 nm laser, but less frequently using the 633 nm laser.

**SERS results for different OPE-4S:AuNP ratios using the 785 nm laser:**


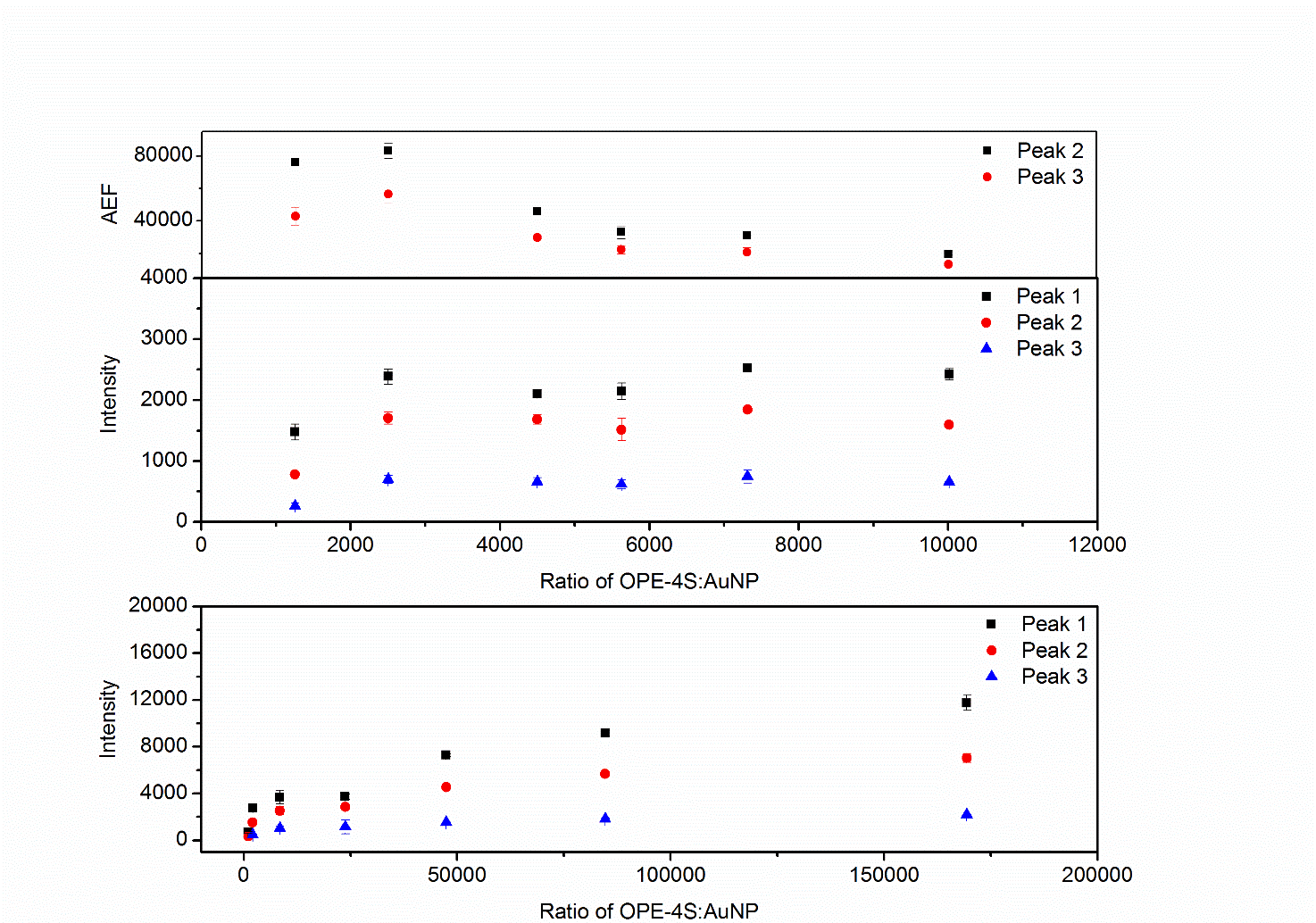


**Figure SI8.** The intensities of the three main peaks in the SERS spectra of OPE-4S in samples containing different OPE-4S:AuNP ratios for two different measurements. The first measurements were performed with a concentration of 18 pM AuNPs, where the AEFs for peak 2 and peak 3 is displayed above (A) and the other with a concentration of 21 pM AuNPs (B). A 785 nm laser was used for the experiments.

The AuNP solution used for Figure SI7A was stored in the fridge and had an AuNP concentration of 18 pM at the time of the measurements. The measurements were performed starting with the smallest ratio and finishing with the highest. The AuNPs used were stored in Eppendorf tubes during the measurements and the possibility that some of the AuNPs adhered to the wall of the tubes cannot be excluded. In Figure SI7B the measurement were performed on 21 pM AuNPs with larger ratios. Here the measurements were performed from the highest to the lowest ratio. Here a slight increase can be seen with increasing OPE-4S:AuNP ratio above 4000, which could be due to the AuNPs sticking to the surface of the Eppendorf tubes.


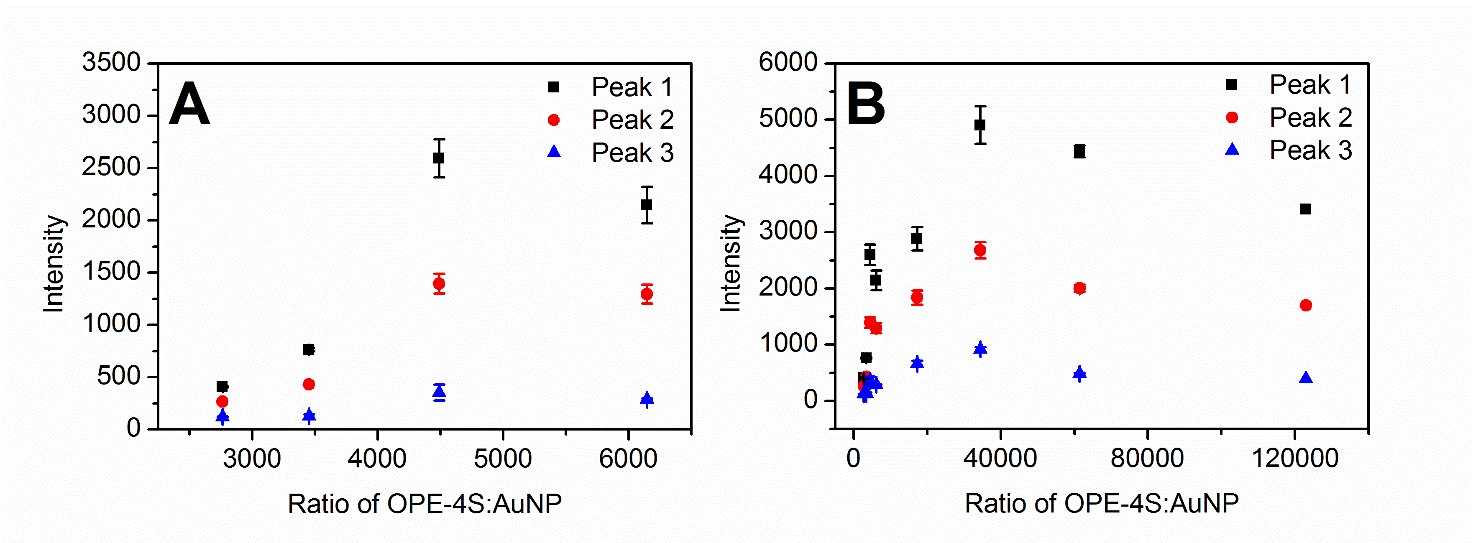


**Figure SI9.** The intensities of the three main peaks in the SERS spectra of OPE-4S in samples containing different OPE-4S:AuNP ratios, with a concentration of 29 pM AuNPs, where a close-up of the small ratio range (A) and the full range (B) is displayed. The excitation source used was 785 nm and the measurements were performed using the Senterra Raman microscope.

The experiment with the 785 nm laser was reproduced on a Senterra Raman microscope. The results are shown in Figure SI8. For both of the experiments with the 785 nm laser the same trends are present as seen for the measurements with the 633 nm laser. From low values to a ratio of about 3000 to 5000, there seems to be a fast increase in the SERS signal. At even larger ratios the SERS signal increases much less with increasing OPE-4S ratio. This behavior will also have an effect on the AEF. In Figure SI7A the AEF corresponding to different samples for peak 2 and 3, is calculated. It is seen that the AEF depends strongly on the ratio. The AEF increases as the ratio is lowered, up until the maximum at the ratio 2500, where it decays again. All the data presented in Figure 6A, Figure SI7 and Figure SI8A shows a steady increase up to a ratio between 2500-5000, followed by a steady or slightly increasing signal after that (up to 200000).

**Individual SERS spectra using the 633 nm laser:**

**
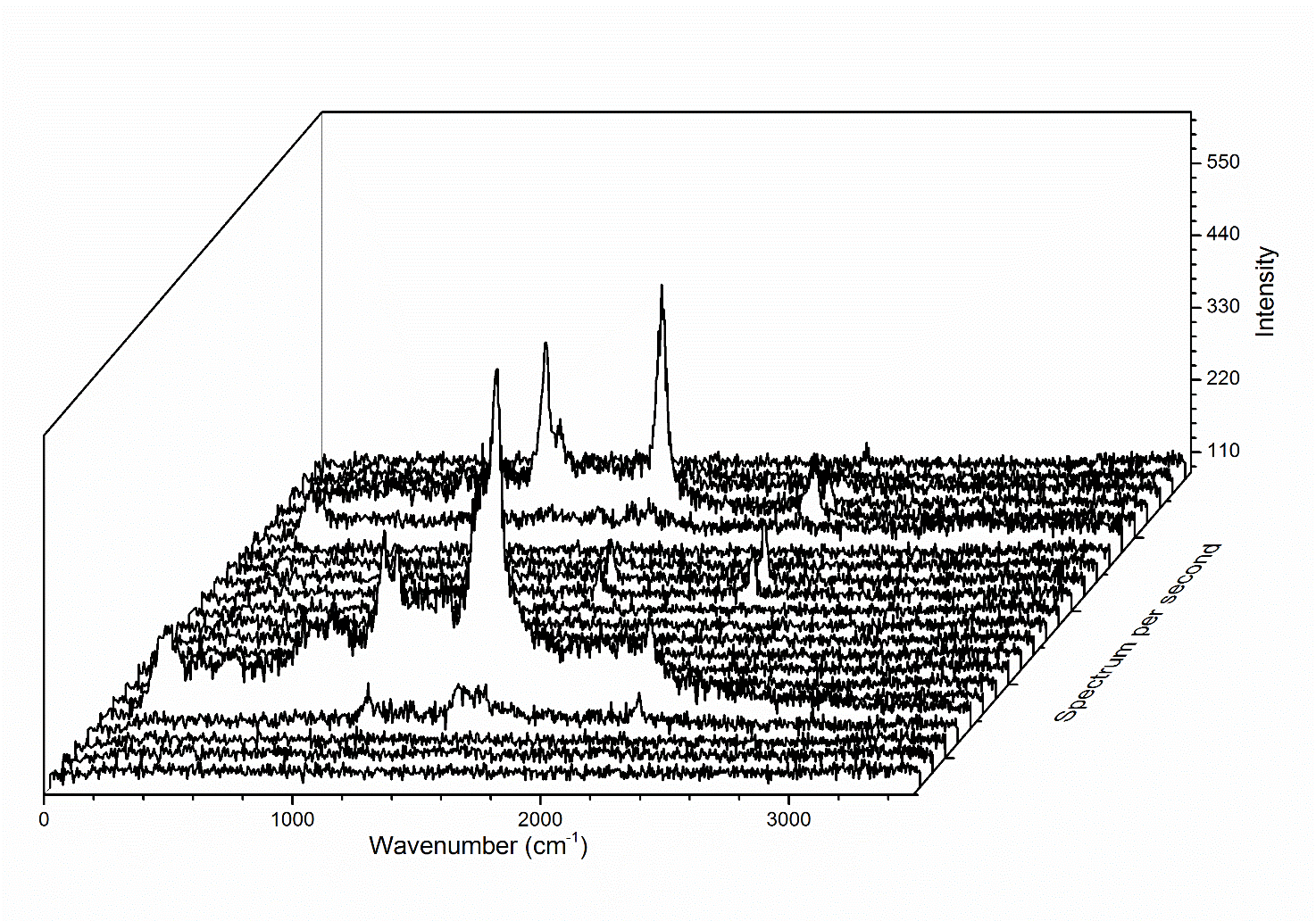
**

**Figure SI10.** Some of the individual SERS spectra out of the 100 measurements of 1 second per spectrum using 25 pM AuNPs and a ratio of 4000 OPE-4S:AuNP.


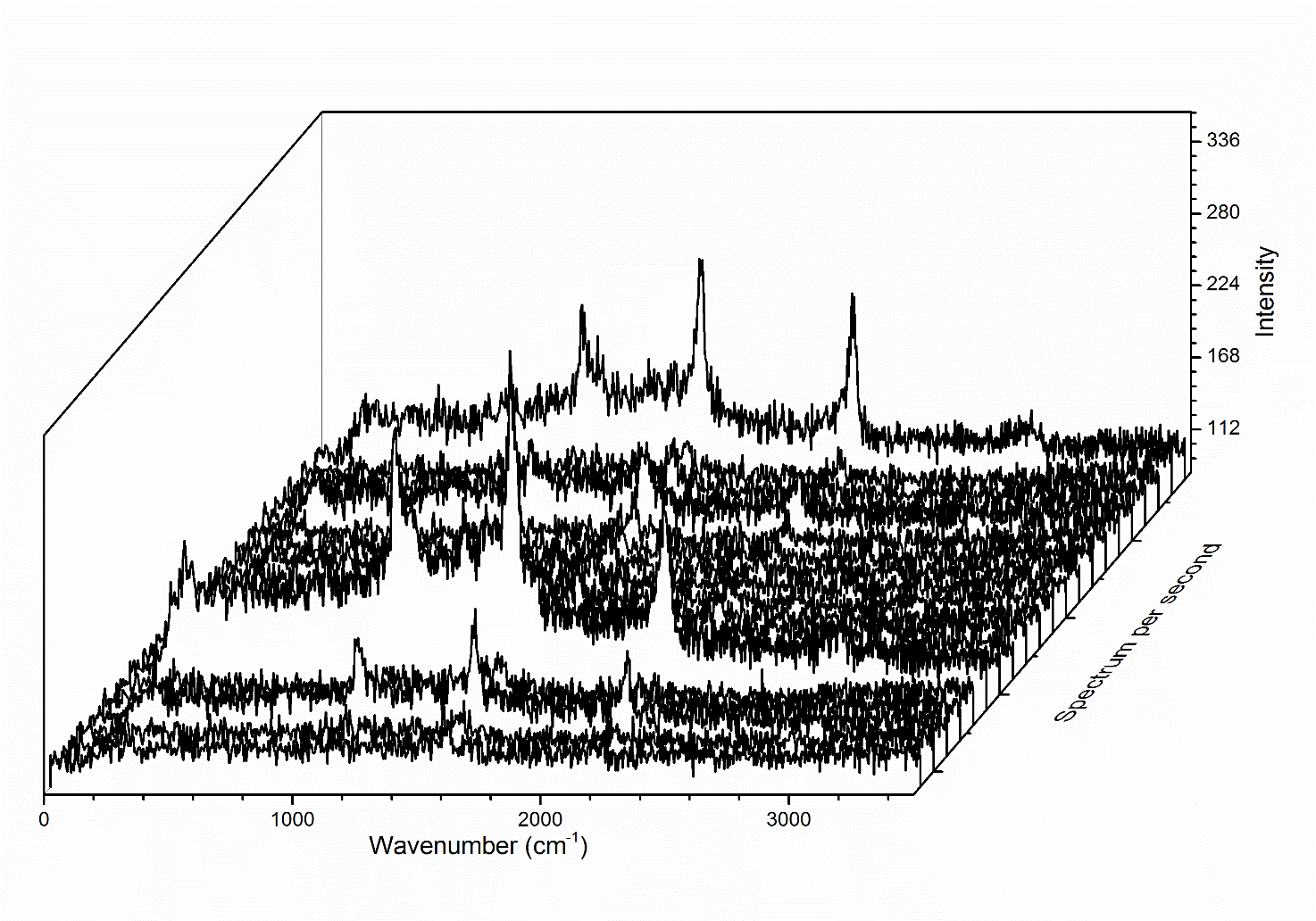


**Figure SI11.** Some of the individual SERS spectra out of the 100 measurements of 1 second per spectrum using 45 pM AuNPs and a ratio of 80000 OPE-4S:AuNP.


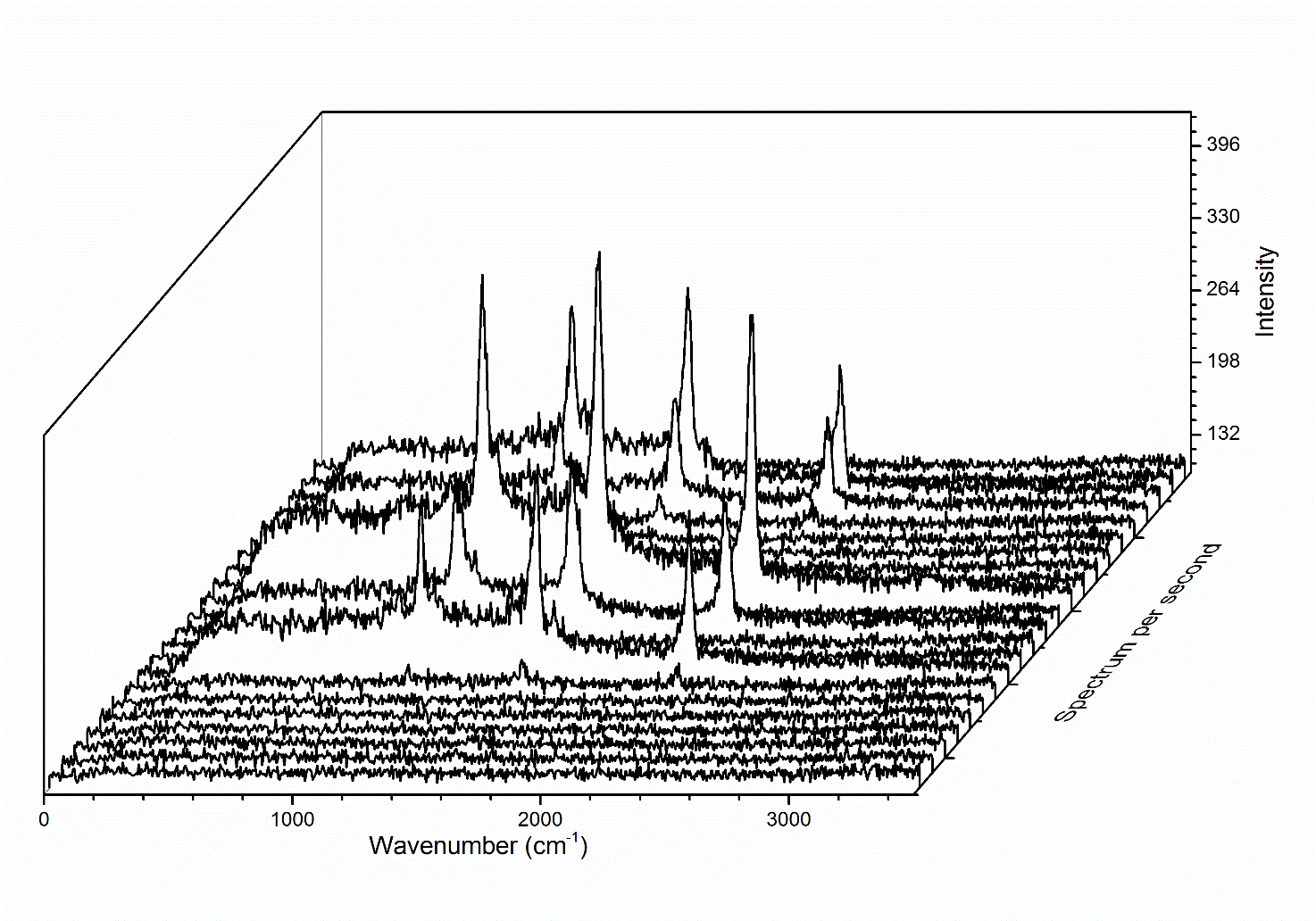


**Figure SI12.** Some of the individual SERS spectra out of the 100 measurements of 1 second per spectrum using 36 pM AuNPs and a ratio of 100000 OPE-2S:AuNP.

1. Haiss, W., et al., *Determination of Size and Concentration of Gold Nanoparticles from UV−Vis Spectra.* Analytical Chemistry, 2007. **79**(11): p. 4215-4221.
